# Long-term bilateral change in pain and sensitivity to high-frequency cutaneous electrical stimulation in healthy subjects depends on stimulus modality: a dermatomal examination

**DOI:** 10.3389/fmed.2023.1337711

**Published:** 2024-01-16

**Authors:** Isabel Escobar-Sánchez, Marta Ríos-León, Julian Taylor

**Affiliations:** ^1^Sensorimotor Function Group, Hospital Nacional de Parapléjicos (SESCAM), Toledo, Spain; ^2^Instituto de Investigación Sanitaria de Castilla-La Mancha (IDISCAM), Toledo, Spain; ^3^Alumna de Doctorado (Ciencias de la Salud), Escuela Internacional de Doctorado, Universidad de Castilla-La Mancha, Ciudad Real, Spain; ^4^Harris Manchester College, University of Oxford, Oxford, United Kingdom

**Keywords:** high-frequency stimulation, long-term potentiation, nociception, pain, sensitivity

## Abstract

**Introduction:**

Contradictory changes in pain and sensitivity at long-term following cutaneous 100 Hz high frequency stimulation (HFS) have been previously observed. Thus, we aimed to document long-lasting changes in multimodal sensitivity following HFS, and factors influencing them.

**Methods:**

Long-lasting changes were assessed with mechanical [brush, von Frey filament (588.2 mN)] and thermal [heat (40°C)/cold (25°C)] bedside sensory testing, and electrical TS (0.2 ms single electrical stimuli), at the homotopic (ipsilateral C6 dermatome), adjacent heterotopic (ipsilateral C5 and C7 dermatomes) and contralateral (contralateral C6 dermatomes) dermatomal sites in a single testing session. TS were applied before and after application of 100 Hz HFS at the ipsilateral C6 dermatome. Subjects rated their sensation and pain intensity to TS, and completed questionnaires related to pain descriptors and quality of life.

**Results:**

Long-lasting changes in mechanical and cold sensitivity was detected up to 45 min after HFS at homotopic C6 dermatome, and a temporary increase in cold sensitivity at 20 min in the contralateral C6 dermatome (*p* < 0.05). A slow development of bilateral depotentiation to electrical pain TS was also detected from 40 min after HFS (*p* < 0.05). Higher HFS-induced mechanical and cold sensitivity was identified in women (*p* < 0.05). Age and quality of life were associated with pain intensity (p < 0.05).

**Conclusion:**

Long-term unilateral and bilateral changes in sensation and pain following electrical HFS have been found. These findings may suggest a new insight into the development of persistent pain mechanisms. Further studies are now needed.

## 1 Introduction

Cutaneous electrical high-frequency stimulation (HFS) induces long-term potentiation (LTP), a neural mechanism of synaptic plasticity that contributes to long-lasting experimental allodynia and hyperalgesia following sensitization of spinal nociceptive pathways (1–5). Indeed, the further study of LTP in patients with clearly defined peripheral or central nervous system injury could lead to a better understanding of the time course and pathophysiology of changes in nociception, and the development of new strategies for the prevention and treatment in acute and chronic pain pathologies (1, 2). More recently widespread changes in nociception and pain have been characterized in patients with unilateral neuropathic pain, including adjacent and contralateral testing sites (6, 7). Information is now required to document rapid changes in multimodal sensitivity at several test dermatomes following cutaneous electrical HFS during the changes in nociception and experimental pain.

LTP-like pain amplification (pain-LTP) has been reported following cutaneous electrical HFS at stimulus intensities sufficient to activate C-fiber nociceptors (2, 3, 8). Indeed, the use of electrode with small contact areas favors the activation of superficial nociceptive Aδ fiber and C fiber (3). Although increased pain intensity has been demonstrated with electrical stimuli applied within the homotopic HFS area (3, 9), reduction in electrical pain perception has also been reported (5, 9–11).

Change in pain sensitivity was also observed outside the HFS site (heterotopic hyperalgesia) as assessed with mechanical pinprick stimuli (1, 3, 11–14). Furthermore, a long-lasting heterotopic increase in painful sensations in response to dynamic mechanical tactile stimuli (3, 10, 13) and heat stimuli were also observed (15). However, other studies have shown that thermal sensitivity remained unaltered after HFS (14). These findings support the hypothesis that homosynaptic and heterosynaptic mechanisms related to LTP could play a role in the development of primary and secondary hyperalgesia (9, 16–18), leading to peripheral and central sensitization to multimodal stimuli in the same and adjacent dermatomes. Finally, HFS-induced pain-LTP responses may also be affected by demographic or psychological factors (13, 19, 20) which may modulate the perception of experimental pain.

The aim of this study was to document long-lasting changes in mechanical, thermal, and electrical test stimuli at adjacent homotopic and heterotopic dermatomal sites following cutaneous electrical HFS, and to identify the impact of demographic and clinical factors on induced experimental pain.

## 2 Materials and methods

This study protocol was approved by the local Clinical Research Ethics Committee (Approval number 149; 2022) and conducted according to the Declaration of Helsinki (21). Participants were recruited from a reference national hospital in Toledo (Spain). All individuals provided written informed consent before their inclusion in the study.

### 2.1 Subjects

Twelve participants aged between 18 and 80 years were recruited (50% women, mean age = 31 ± 15; Table 1). The exclusion criteria were history of chronic pain or/and neurological or psychiatric diseases, peripheral nervous system injury or neuropathy, diseases causing potential neural damage (e.g., diabetes, diseases of the immune system, oncological diseases), previous clinical history of cervical surgery, injuries or surgery affecting the upper limb, altered sensitivity in the tested regions, frequent headaches, and/or orofacial pain.

**Table 1:** Baseline demographic and clinical characteristics of healthy participants (*n* = 12).

Subject number	Sex	Age (years)	BMI	EQ-5D-5L (VAS)	EQ-5D-5L (index)	PHQ-9	GAD-7
#1	M	57	32.1	90	1	0	2
#2	M	24	27.3	95	1	11	5
#3	F	43	19.9	70	1	6	4
#4	F	20	21.3	95	1	2	1
#5	F	22	26.0	95	1	5	9
#6	F	30	22.3	95	0.80	4	10
#7	F	21	23.3	80	1	4	5
#8	M	30	23.4	100	1	3	0
#9	M	21	21.1	95	1	4	1
#10	M	22	23.3	95	1	4	1
#11	F	63	17.0	95	0.80	0	1
#12	M	19	25.3	70	0.74	5	7
Total (*n* = 12)	6 M 6F	31 ± 15.1	23.6 ± 3.9	89.6 ± 10.3	0.94 ± 0.1	4 ± 2.9	3.8 ± 3.4

BMI, body mass index; EQ-5D-5L, the five-level version of EuroQol-5D; F, female; GAD-7, the 7-item generalized anxiety disorder (GAD) scale; M, male; PHQ-9, the Patient Health Questionnaire-9; VAS, visual analogue scale.

### 2.2 Experimental protocol

Each subject was invited to participate in a single testing session that lasted approximately 1 h. Sensory testing was conducted in a quiet indoor laboratory at ambient temperature (22°C–26°C). The subjects sat in a comfortable chair and were familiarized with the experimental procedure. Participants were prohibited from undertaking vigorous physical activities or drinking caffeinated beverages at least 24 h before the experiment.

**Figure 1:**
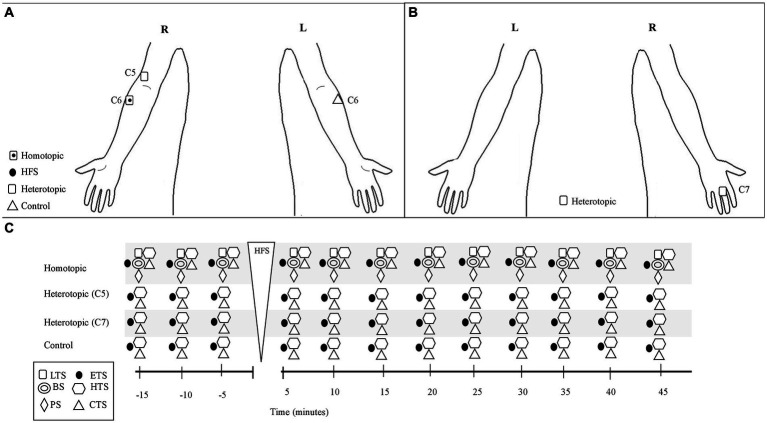
Schematic of the 100 Hz-HFS experimental test protocol and stimulation location of the test and conditioning stimuli. **(A)** Frontal plane (anterior): anatomical locations for application of the test stimuli and conditioning stimulus (HFS). **(B)** Frontal plane (posterior): anatomical locations for the test stimuli. **(C)** Schematic of the experimental protocol. The test stimuli were applied at 5 min intervals before and up to 45 min after the electrical HFS, which was applied for 60 s at the C6 dermatome on the dominant arm. Test stimuli were applied at the C6 (homotopic), C5, and C7 (heterotopic) dermatomes on the dominant arm, and at the contralateral C6 dermatome at 5 min intervals. BS, brush stimulus; CTS, cold test stimulus; ETS, electrical test stimulus; HTS, heat test stimulus; LTS, light touch stimulus; PS, pinprick stimulus; R, right; L, left.

The session started with baseline testing with stimuli (TS, see below) applied at the C5, C6, and C7 dermatomes at the dominant side, and at the contralateral C6 dermatome at 5 min intervals for 15 min (see Figure 1 and TS section below). The cutaneous electrical HFS was then applied to the C6 dermatome at the dominant side during 60 s (see HFS section below), and the subject was asked to rate the electrical pain intensity during the electrical stimulus. After HFS, TS were applied to the C5, C6, and C7 dermatomes at the dominant side, and at the contralateral C6 dermatome to detect homotopic and heterotopic effects at 5 min intervals for 45 min. Thus, the study compared post HFS effects on sensory and pain intensity with the 15 min baseline recordings made from the same participant. Subjects were blinded to the study hypothesis.

#### 2.2.1 High frequency stimulation (HFS)

Electrical stimuli were applied through a bipolar concentric electrode (522100, BCS-probe 90 mm straight, Inomed Medizintechnik GmbH, Emmendingen, Germany; diameter, 2 mm) on the dominant forearm, 5 cm distal to the cubital fossa (C6 dermatome) at the dominant side (conditioned site, Figure 1), using a constant current stimulator (DS7A, Digitimer Ltd., Hertfordshire, United Kingdom) (1, 3). The small diameter of the electrode permitted delivery of a high current density achieved at low stimulus intensities, which favors activation of superficial nociceptive Aδ fiber and C fiber afferents (3). Stimulus intensity was adjusted at which the electrical stimulus induced a pain rating of 4/10 (“Pain-4”) for each participant with the 11-point numerical rating scale (NRS; 0: no pain, 10: maximum pain) (22): prior to electrical TS and CS, “Pain-4” was assessed and determined by applying single electrical pulses (see below). In this sample, intensity for “Pain-4” corresponded to 10 times the electrical detection threshold (DTh) (1–4, 10, 13, 14, 23, 24) (mean intensity: 1.2 ± 0.8 mA).

The cutaneous electrical HFS was applied as a 1 s train of five pulses applied at 100 Hz (2 ms pulse width), repeated every 10 s inter-train intervals for 1 min, to induce pain-LTP (1, 3). The participants reported the HFS pain intensity with the NRS (0: no pain; 10: maximum pain) at 10 s intervals.

If the perceived pain became unbearable, subjects were allowed to remove their arm from the stimulating electrode at any time.

#### 2.2.2 Test stimuli (TS)

Changes in sensory and pain sensitivity were tested using three different test stimuli (TS): electrical, mechanical, and thermal TS. The TS were applied at the forearm 5 cm distal to the cubital fossa corresponding to the C6 dermatome (3) at the dominant (homotopic site) and nondominant (contralateral C6 dermatome, control site) sides (Figure 1). At the dominant side, TS were also applied to the lateral (radial) side of the antecubital fossa just proximal to the elbow and dorsal surface of the proximal phalanx of the middle finger corresponding to the C5 and C7 dermatomes (heterotopic sites, Figure 1), respectively, according to the American Spinal Injury Association Impairment Scale Key Sensory Point (25). These TS sites were selected to identify local spinal modulatory effects following the HFS.

##### 2.2.2.1 Electrical TS

Electrical TS were applied with the bipolar concentric electrode (522100, BCS-probe 90 mm straight, Inomed Medizintechnik GmbH, Emmendingen, Germany; 2 mm tip diameter) applied over the selected dermatomal test sites with a constant current stimulator (DS7A, Digitimer Ltd., Hertfordshire, United Kingdom) (3). The electrical TS was presented as 3 pulses (2 ms pulse width) applied at 0.2 Hz for repeated at 5 min intervals during 15 min prior to HFS. The electrical TS were applied at “Pain-4” intensity as assessed previously for each participant. The intensity for DTh, electrical pain threshold, and “Pain-4” (10 x DTh) were assessed and determined by applying single electrical pulses steps of 0.1 mA until the subjects reported electrical sensation, pain, and a pain rating of 4/10, respectively. Additionally, each subject was familiarized with the electrical pulses prior to assessment of electrical pain threshold.

For electrical TS, the subjects were instructed to assess the unpleasantness of the sensation as well as pain intensity using the NRS (0: no pain/discomfort; 10: maximum pain/discomfort) at 5 min intervals, before and up to 45 min after the HFS (see Figure 1). Thus, these assessments were performed at 5, 10, and 15 min before the HFS, and 5, 10, 15, 20, 25, 30, 35, 40, and 45 min after the HFS (Figure 1).

##### 2.2.2.2 Mechanical and thermal TS

Mechanical TS were applied using a cotton bud (Q-tip, United States), brush (SenseLab 05, Somedic AB, Sweden), and a von Frey filament (Stoelting Co., United States; No. 5.88, nominal buckling force 588.2 mN) applied at the test sites to assess dynamic mechanical detection sensitivity (light touch sensation), dynamic mechanical allodynia (brushing), and mechanical pain sensitivity (pinprick), respectively. The sensation perceived with each mechanical stimuli at each test area was compared by the participant to the V2 dermatome (zygomatic bone) corresponding to the AIS Key Sensory Point (25) as an additional control area. The cotton bud was applied as one stroke (1–2 cm length) and the brush as 4 strokes (1–2 cm length) (1). The von Frey filament was placed perpendicularly to the tested surface for 1–2 s until it bent, and then kept in place for 2 additional seconds before the stimulus was removed (26). Aβ-fiber low-threshold mechanoreceptors and Aδ fiber nociceptors are predominantly activated by these mechanical TS (14).

Finally, thermal TS were applied using thermal rollers (Somedic SenseLab AB, Sösdala, Sweden; Roll-Temp II) at the test sites as strokes of 5 cm length to determine heat and cold sensitivity. The thermal rollers apply heat at 40°C and cold at 25°C. Aδ- and C-fiber afferents are predominantly activated by these thermal TS (14).

For thermal and mechanical TS, the subjects reported the perception at the test sites as compared to the V2 dermatome control area using a scale from 1 to 3 [i.e., (1) TS perceived as less intense than V2, (2) TS perceived as same intensity as V2, (3) TS perceived as more intense than V2]. This scale was adapted from validated scales used for standardized sensory examinations (27, 28). In addition, the pain intensity of the TS was assessed using the NRS (0: no pain; 10: maximum pain). Both sensory perception and pain intensity to TS were assessed at 5-min intervals before and up to 45 min after the HFS (Figure 1). Thus, these assessments were performed at 5, 10, and 15 min before the HFS, and 5, 10, 15, 20, 25, 30, 35, 40, and 45 min after the HFS (Figure 1).

### 2.3 Neuropathic sensations

The presence of neuropathic characteristics related to pain and induced by HFS was assessed with the seven items of the DN4 questionnaire: burning, painful cold, electric shocks, tingling, pins and needles, numbness, and itching (29). The participants rated the intensity of every neuropathic sensation using the NRS (0: absence; 10: maximum intensity) before and up to 45 min after the HFS. Thus, these assessments were performed at 5, 10, and 15 min before the HFS, and 5, 10, 15, 20, 25, 30, 35, 40, and 45 min after the HFS.

### 2.4 Levels of anxiety and depression

The Patient Health Questionnaire-9 (PHQ-9) and the 7-item generalized anxiety disorder (GAD) scale (GAD-7) were self-report instruments used to assess depression and anxiety, respectively, at baseline.

The PHQ-9 is a self-report screening test that includes nine items related to diagnostic criteria for major depression. The presence of depressed mood, anhedonia, sleep problems, feelings of tiredness, changes in appetite or weight, feelings of guilt or worthlessness, difficulty concentrating, feelings of sluggishness or worry, and suicidal ideation over the previous 2 weeks period were assessed with this questionnaire. Items are scored on a four-point Likert scale from 0 (never) to 3 (most days). Thus, total severity scores range from 0 to 27 points indicating moderate (10–14 points), moderately severe (15–19 points) and severe (20–27 points) levels of depressive symptoms (30, 31).

The GAD-7 includes 7 items related to anxiety symptoms rated on a 4-point response scale ranging from 0 (“never”) to 3 (“nearly every day”). The total severity score ranges from 0 to 21 points indicating minimum (0–4), mild (5–9), moderate (10–14) and severe (15–21) anxiety (32, 33).

### 2.5 Perceived quality of life

Health-related quality of life was assessed with the paper-based five-level version of EuroQol-5D (EQ-5D-5L), which assesses independent daily activities, mobility, self-care, perceived pain, and depression/anxiety impact domains. All responses, ranging from 1 (absence of problems) to 3 (severe problems), were converted into a single index number, which corresponds to the health state according to standardized values ranging from 0 (health state equivalent to death) to 1 (optimal health) (34, 35). Additionally, this questionnaire also includes a visual analogue scale, where general health during the previous 24 h is rated on a scale from 0 (worse imaginable health) to 100 (best imaginable health) (35). This assessment was performed at baseline.

### 2.6 Statistical analysis

Data analysis was conducted using Sigma Plot version 11.0 (Systat Software, Inc., United States) and SPSS version 22.0 (IBM Corp., Armonk, New York, United States). Results are expressed as mean, standard deviation (SD), and standard error (SE). In general, the Shapiro–Wilk test revealed that quantitative data, except for TS sensitivity, followed a normal distribution (*p* > 0.05). Measures of TS sensitivity were transformed into percentages; the scores of 1 to 3 were transformed to 33%, 66% and 100%, respectively. The repeated measures analysis of variance (RM-ANOVA) test followed by post-hoc pairwise tests using the Holm-Sidak correction was performed in order to investigate change in TS sensation, pain intensity following HFS, or pain intensity during HFS (trend of pain across time). General differences for TS sensations or pain intensity, and neuropathic sensations before and after HFS were calculated with the paired student’s *t*-test. The interaction between dermatomal test site and time after HFS on TS sensation or pain intensity was analyzed with the two-way ANOVA. Additionally, the possible influence of baseline demographic or clinical characteristics on HFS-induced changes in sensation or pain intensity were analyzed with the *χ*^2^ tests of independence, unpaired student *t*-test, and one-way ANOVA followed by post-hoc pairwise tests to control for multiple comparisons. Finally, the Pearson correlation (*r*) test was used to evaluate associations between variables. The statistical analysis was conducted at a 95% confidence level with a *p*-value <0.05 which was considered statistically significant.

## 3 Results

### 3.1 Demographic and clinical data of the participants

Twelve healthy subjects (50% women, mean age = 31 ± 15 years) were recruited. Clinical and demographic data are presented in Table 1. No significant differences in baseline characteristics were identified for age or sex, except for body mass index (BMI; *p* = 0.034): overweight individuals (BMI: 25.0–29.9) revealed higher PHQ-9 scores compared to normal weight (BMI 18.5–24.9) (7 ± 3.5 VS 3.86 ± 1.2).

### 3.2 Perception of CS

**Figure 2:**
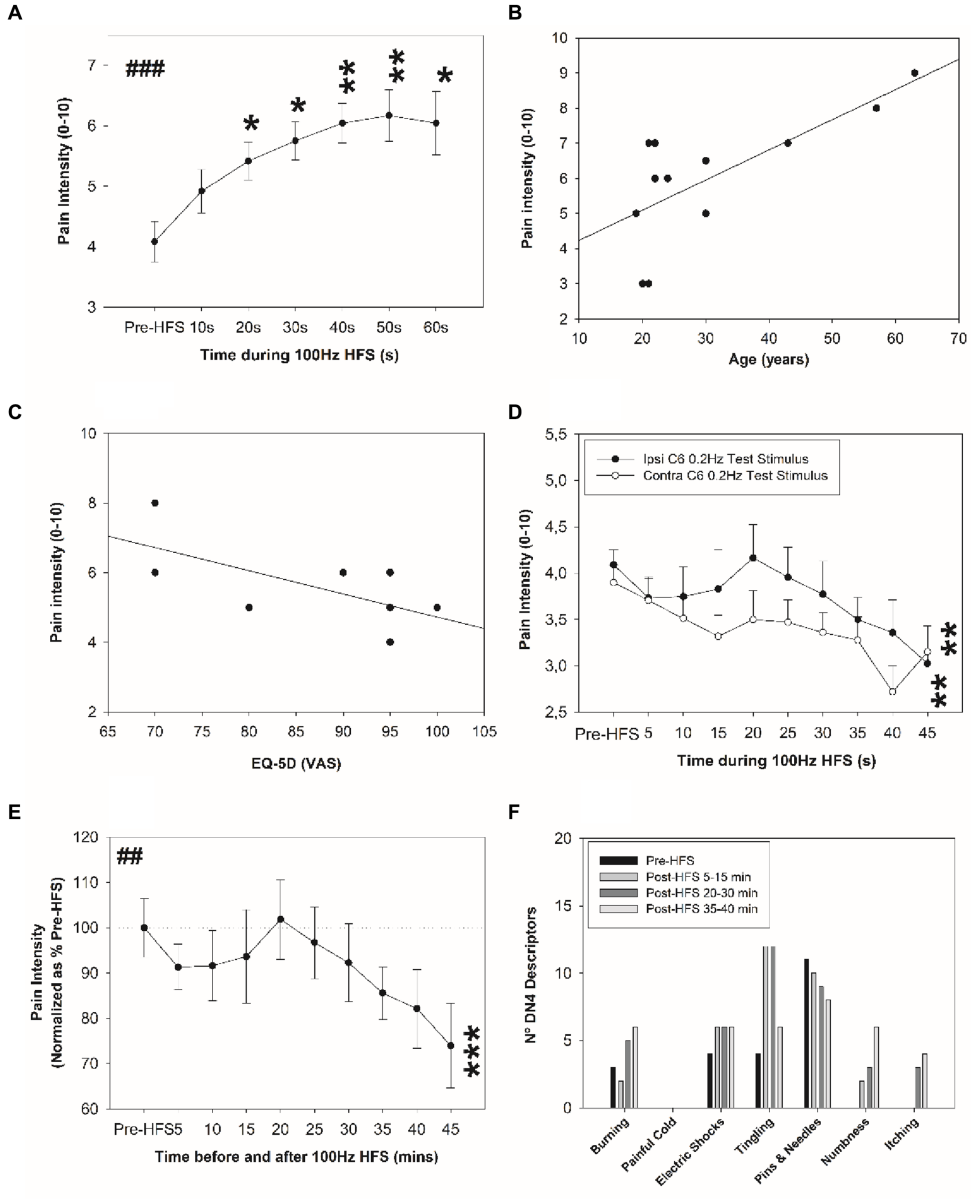
Change in electrical pain sensitivity after HFS. **(A)** Pain ratings perceived during HFS. Correlation between perceived pain intensity during the HFS and **(B)** age (years), and **(C)** health-related quality of life. **(D)** Perceived pain intensity elicited by the single electrical stimuli before and after HFS at the homotopic ipsilateral C6 dermatome site and at corresponding site in the contralateral arm. **(E)** Normalized perceived pain intensity elicited by the single electrical stimuli before and after HFS at the homotopic stimulation site. **(F)** Frequency of neuropathic sensations experienced before and after HFS. *p < 0.05, **p < 0.01, ##*p* < 0.01, ***p < 0.001 (RM-ANOVA ##, *post-hoc* pairwise tests *). Data presented as mean and standard errors.

No visible skin injuries occurred following the electrical HFS, although skin blushing under the stimulated area was observed. Figure 2A shows pain ratings perceived during the HFS. The 100 Hz conditioning stimulation induced an increasing pain intensity during the HFS (temporal summation), i.e., when compared to the initial pain intensity rated with HFS value, a significant increase in pain intensity was perceived from 20 s up to 60 s.

### 3.3 Pain perception associated with electrical HFS: impact of age and quality of life

Figure 2A shows the mean (and SE) perceived pain intensity elicited during the HFS at ipsilateral C6 dermatome (dominant side; see Figure 1A). A significant increase in pain intensity was observed from 20 to 60 s (Figure 2A). The pain intensity perceived during the electrical HFS was significantly and positively associated with age (*r* = 0.716; *p* = 0.009): older individuals reported higher pain intensity during the electrical stimulus (Figure 2B). Individuals older than 40 years old perceived higher pain intensity during HFS compared to those subjects younger than 30 years old (8 ± 1 vs. 4.5 ± 1.9, *p* = 0.02). Additionally, a significant negative association was observed between pain intensity during HFS and perceived quality of life (*r* = −0.633; *p* = 0.027): subjects who reported poor quality of life perceived higher pain intensity with HFS (Figure 2C). No other significant differences in age range were found (*p* > 0.05) and no significant differences in BMI were revealed (*p* > 0.05).

### 3.4 Electrical HFS-induced changes in electrical pain sensitivity

Single electrical test applied stimuli at the ipsilateral C6 and contralateral C6 dermatomal test sites before and after electrical HFS revealed significant depotentiation of pain intensity (Figure 2D). A main effect of time was revealed: a significant decrease in perceived pain intensity was identified for the ipsilateral C6 dermatomes at 45 min and contralateral C6 test site at 45 min after HFS (*p* < 0.01). No significant potentiation was observed at 20 min after HFS for the ipsilateral test site. The percentage change in ipsilateral C6 pain intensity at 45 min after HFS is shown in Figure 2E.

Table 2 shows changes in electrical pain sensitivity reported by each participant at 5–15, 20–30 and 35–45 min after HFS: 75% of healthy individuals showed depotentiation between 35–45 min after HFS; however, 50% of participants demonstrated potentiation at 20–30 min interval after HFS.

**Table 2:** Change in individual electrical sensitivity during 100 Hz-HFS-induced potentiation or depotentiation presented as a percentage of the cohort pre-HFS pain intensity (*n* = 12).

Participant number	% pain modulation between 5–15 min	% pain modulation between 20–30 min	% pain modulation between 35–45 min
1	+48	+34	+34
2	+25	+26	-10
3	−49	−39	−51
4	−6	+7	+3
5	−3	−3	−3
6	−17	−46	−57
7	−31	−20	−40
8	−19	−14	−6
9	−21	−30	−58
10	+11	+70	+11
11	0	+23	−12
12	0	+3	−17
Potentiation (mean ± SE) and % of cohort presenting depotentiation	28.0 ± 10.8 (25)	32.0 ± 10.5 (50)	16.0 ± 9.3 (25)
Depotentiation (mean ± SE) and % of cohort presenting depotentiation	−20.9 ± 5.9 (58.3)	−25.3 ± 6.7 (50)	−29.6 ± 8.5 (75)

HFS, high-frequency stimulation; SE, standard error. The individual 100 Hz-HFS-induced potentiation or depotentiation was calculated as a percentage of the pre-HFS cohort normalized electrical pain intensity.

### 3.5 Effect of electrical HFS on induced neuropathic sensations

During the 45 min after HFS, a significant increase in the number of neuropathic sensations were reported (0.6 ± 0.7 vs. 1.0 ± 0.8; *p* = 0.023), and especially when measured between 15 and 45 min after HFS (0.6 ± 0.7 vs. 1.0 ± 0.9; *p* = 0.018). The greatest change in frequency of neuropathic sensations was associated with tingling sensation: after HFS, the presence of tingling sensation tripled when compared in the pre-HFS interval (Figure 2F). No other significant changes were found.

### 3.6 Changes in mechanical and thermal sensitivity after HFS

**Figure 3:**
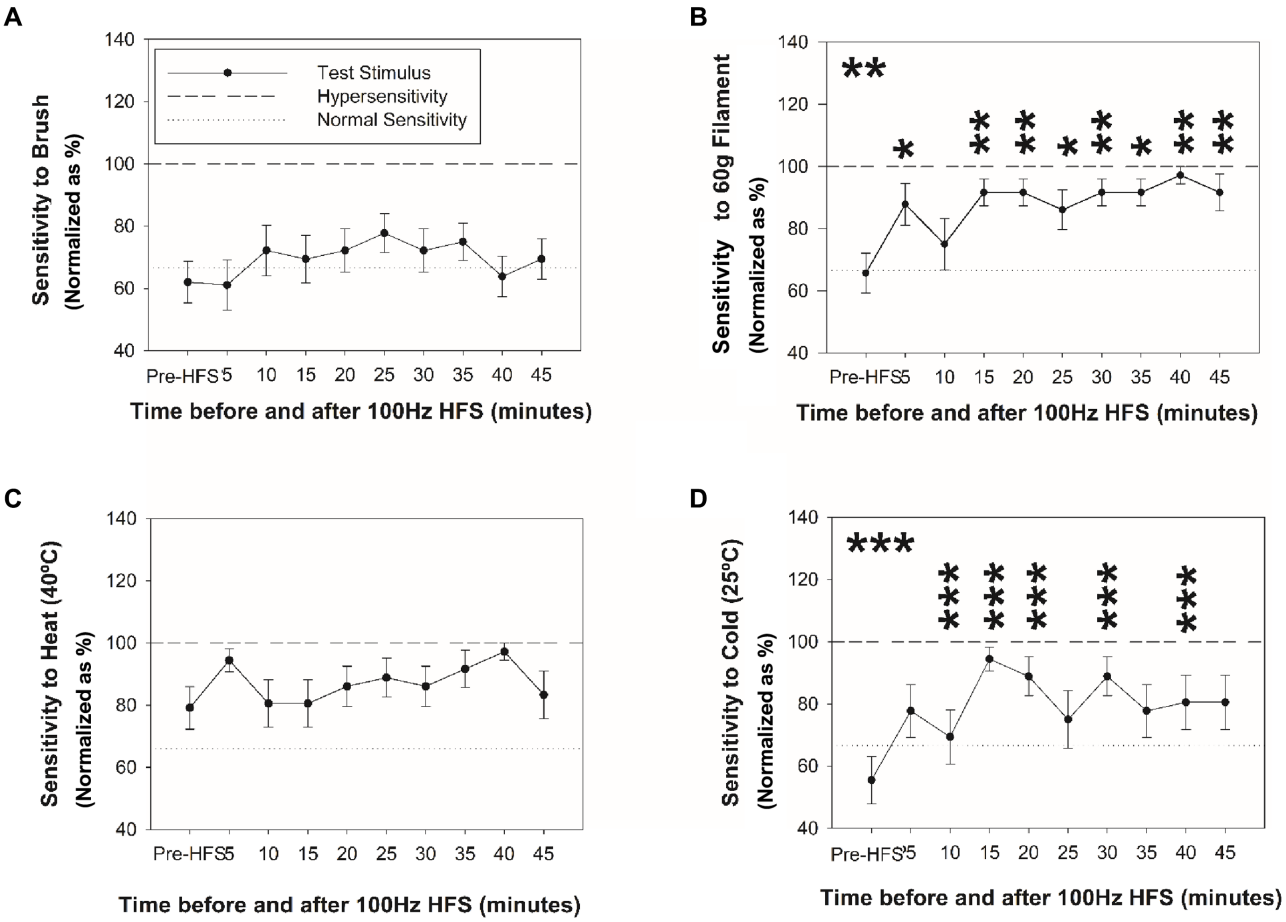
Changes in mechanical and thermal sensitivity after HFS. Sensitivity at the ipsilateral homotopic C6 dermatome before and after HFS to brush **(A)**, 60 g von Frey filament **(B)**, heat **(C)**, and cold stimuli **(D)**. *p < 0.05, **p < 0.001, ***p < 0.001 (RM-ANOVA, *post-hoc* pairwise tests). Data presented as mean and standard errors.

Figure 3 reveals changes in mechanical sensitivity after HFS at the ipsilateral C6 dermatome test site (dominant side). No change in sensitivity was identified with brush (Figure 3A). In contrast, a significant increase in mechanical pain sensitivity (von Frey filament) at ipsilateral C6 dermatome (Figure 3B) was identified from 5 and up to 45 min after HFS. No other significant changes or significant differences among sites were found.

**Figure 4:**
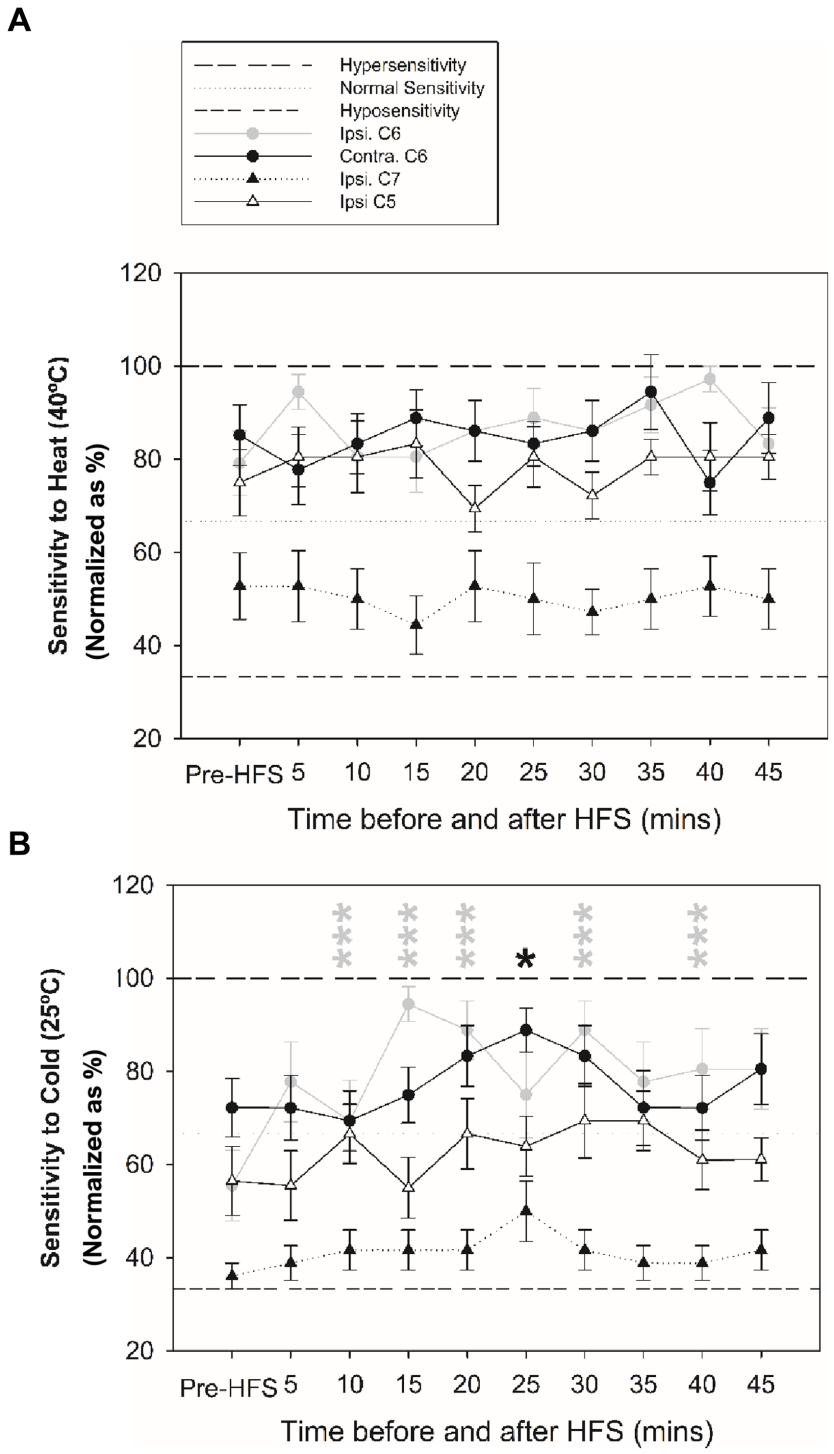
Changes in thermal sensitivity at ipsilateral C5, C6 and C7 dermatomes, and contralateral C6 test site before and after HFS. **(A)** Sensitivity to heat at ipsilateral before and after HFS. **(B)** Sensitivity to cold before and after HFS. *p < 0.05, ***p < 0.001 (RM-ANOVA, post-hoc pairwise tests). Data presented as mean and standard errors.

Figures 3, 4 reveal changes in thermal sensitivity after HFS at ipsilateral C6 and C7 dermatomes, and contralateral C6 dermatome. For cold sensitivity (Figure 3C), a significant main effect of time was revealed: a significant increase in cold sensitivity was found at the ipsilateral C6 dermatomes from 10 up to 40 min after HFS (Figure 3D). In addition, an increase in contralateral cold sensitivity at the C6 dermatome at 25 min after HFS was also identified. Additionally, some individuals also presented low pain intensity (NRS: 1–3) related to cold hypersensitivity after HFS. No other significant changes were found in the ipsilateral C5 or C7 dermatomes.

### 3.7 Sex differences in sensitivity and pain intensity after HFS

Significant sex differences were found in cold sensitivity induced after HFS at C6 dermatome (dominant side), particularly between 15 and 45 min (*p* = 0.02): women showed higher cold sensitivity than men (3.0 ± 0.0 vs. 2.5 ± 0.8). Additionally, significant sex differences were found in mechanical pain sensitivity (pinprick) between 5 and 45 min after HFS at the C6 dermatome (dominant side, *p* = 0.045), particularly between 15–45 min (*p* = 0.036): women showed higher mechanical pain sensitivity than men (0.2 ± 0.04 vs. 0.0 ± 0.0). No other significant sex differences were found and no significant associations with levels of anxiety or depression were revealed.

## 4 Discussion

Although the small sample size needs to be considered in the interpretation of the current findings, this study reveals for the first time simultaneous and significant long-lasting changes in cold sensitivity at the conditioned site and at the contralateral test site after electrical HFS. In addition, a possible sex and age effect on the perception of HFS pain and induced long-lasting changes in thermal and mechanical sensitivity were shown in healthy subjects. Importantly the use of electrical test stimuli sufficient to activate high-threshold afferent fibers has revealed a late development of pain depotentiation in electrical pain sensitivity at both the conditioned and contralateral test sites. Identification of a spectrum of long-term potentiation and depotentiation of multi-modal stimuli determines the need to better define both the conditioning and test stimuli parameters for the assessment of modulatory pain mechanisms.

### 4.1 HFS induces neuropathic sensations and long-lasting mechanical hyperalgesia

Electrical HFS increased the perception of neuropathic sensations, especially tingling as the most common neuropathic descriptor. Previously found hot and burning sensations were reported as the most common neuropathic descriptors (23), which were related to perceived pain intensity at the HFS site. These sensations have been attributed to the activation of Aδ-fibers and C-fibers (23).

This study also showed a significant increase in mechanical pain sensitivity at the HFS-conditioned site supporting similar findings observed around the HFS-conditioned site in previous studies (2, 3, 8), possibly mediated by Aδ fibers (3, 10, 13) and primary nociceptive C-fibers (3, 4, 12, 23, 36). Furthermore, long-lasting mechanical hypersensitivity to pinprick stimuli for more than 45 min has been reported previously (2), with an increase in pinprick-evoked brain potentials (37). Although the present findings should be considered with caution due to the small sample size, the results of the current study support the involvement of LTP in the mechanical sensitization at the conditioned site following electrical HFS.

After HFS, significant sex differences in mechanical and cold pain sensitivity were also revealed for the first time: women showed higher mechanical and cold pain sensitivity than in men. A previous study found no significant sex differences on HFS-induced pain sensitivity (13), although women showed a greater increase in mechanical pain sensitivity after electrical stimulation (13) and in pain sensitivity in general (38). Although the present findings need to be considered with caution due to the small sample size and further studies are needed to understand possible mechanisms underlying sex differences, pain perception during HFS could help to identify risk factors which underlie the development of chronic pain (39). This includes the possible association of pain perception during HFS with age and health-related quality of life.

### 4.2 HFS induces bilateral long-lasting sensitivity to cold stimuli

The current findings revealed a significant increase in cold sensitivity in both the ipsilateral and contralateral C6 dermatomes after HFS, unlike previous studies that reported no significant changes (14). The significant increase in cold sensitivity at the conditioned site could indicate a possible role for Aδ-fibers in cold sensitivity and hyperalgesia (40, 41) and thermal hyperalgesia at the stimulated area is a typical feature of primary hyperalgesia associated with this sensitization (4, 42). The observation of cold sensitivity in the contralateral dermatome could be explained that lumbar Lamina I neurons also receive contralateral afferent information (43), which may be disinhibited following periods of intense pain stimulation such as that applied with the electrical HFS stimulus (6, 7). This mechanism would lead to contralateral sensory information to projection neurons contributing to thermal hypersensitivity and mirror-image pain. Indeed, observations of cold hyperalgesia in participants with unilateral carpal tunnel syndrome have been observed (44) and quantitative sensory testing has revealed evidence for contralateral changes in sensitivity with different pain pathologies (6, 7). Similarly, HFS could also trigger LTP of unconditioned C-fiber synapses through glial cell processes (16). After HFS, no significant change in heat sensitivity was revealed supporting previous studies (2, 4, 14, 45). Nevertheless, the present findings should be considered with caution due to the small sample size.

### 4.3 HFS-induces long-term depotentiation of electrical pain intensity

The current study also found a significant depotentiation in electrical pain sensitivity at the conditioned and contralateral sites, supporting a concurrent reduction of the perceived pain intensity after HFS at the conditioned site and several remote test sites (5, 9–11), including the contralateral test area (5, 9, 10). Initial studies of HFS on electrical test stimuli revealed a long-term potentiation of pain intensity elicited by single electrical stimuli at the HFS site (1–3, 8, 13).

HFS could activate hypoesthesia induced by continuous electrical stimulation at C-fiber strength (4, 46). HFS could also reverse LTP with the induction of long-term depression (LTD) or LTP-induced depotentiation, depending on neuronal properties such as the membrane potential of spinal dorsal horn neurons (9), the type of cell activated demonstrated as LTP in spinothalamic neurons and LTD at GABAergic neurons (2, 47), and finally the implication of glia (48). Although the present findings should be considered with caution due to the small sample size, the recent observations of inhibition of electrical pain intensity at several test sites after HFS suggest that careful selection of test stimulus modality is critical to understanding processes of LTP and LTP-induced depotentiation in pain modulation.

### 4.4 Limitations

The small participant sample size requires some caution to be made in the interpretation and generalization of results from the present study. Nevertheless, sample size was based on sample sizes used in previous studies (3, 14), which even included smaller sample sizes (3). Furthermore, to our knowledge, this is the first study that has identified long-lasting changes in cold sensitivity after HFS at the conditioned site and contralateral test site. Further study of cold sensitization following HFS using quantitative sensory testing protocols may provide an important insight into the development of persistent pain mechanisms. In addition, the association of pain intensity experienced during HFS with age and quality of life, and the sex effect of induced pain sensitivity after HFS warrants further investigation into the influence of these demographic and clinical characteristics on LTP and pain sensitization in humans.

## 5 Conclusion

The present study revealed a significant long-lasting change in thermal, mechanical, and electrical sensitivity after HFS. These effects included a significant increase in mechanical pain sensitivity at the HFS-conditioned site, in addition to an increase in cold sensitivity and depotentiation in electrical pain sensitivity at both the ipsilateral and contralateral test dermatomes. In addition, changes in mechanical and cold sensitivity after HFS were dependent on sex. These results could lead to a better understanding of pathophysiological mechanisms in chronic pain pathologies in order to identify risk factors and to develop new management and prevention strategies for chronic pain. However, these results need to be considered with caution due to the small sample size. Future studies with larger sample sizes and chronic pain conditions are now required to confirm the current findings.

## Data availability statement

The original contributions presented in the study are included in the article/supplementary material, further inquiries can be directed to the corresponding author.

## Ethics statement

This study was approved by the local Clinical Research Ethics Committee (Approval number 149; 2022). This study was conducted in accordance with the local legislation and institutional requirements. The participants provided their written informed consent to participate in this study.

## Author contributions

IE-S: Conceptualization, Data curation, Formal analysis, Investigation, Methodology, Resources, Software, Validation, Visualization, Writing – original draft, Writing – review & editing. MR-L: Conceptualization, Data curation, Formal analysis, Investigation, Methodology, Resources, Software, Supervision, Validation, Visualization, Writing – original draft, Writing – review & editing. JT: Conceptualization, Formal analysis, Funding acquisition, Investigation, Methodology, Project administration, Resources, Supervision, Validation, Visualization, Writing – review & editing.

## References

[1] Pfau DB Klein T Putzer D Pogatzki-Zahn EM Treede RD Magerl W. Analysis of hyperalgesia time courses in humans after painful electrical high-frequency stimulation identifies a possible transition from early to late LTP-like pain plasticity. Pain. (2011) 152:1532–9. doi: 10.1016/j.pain.2011.02.037, PMID: 21440369

[2] Xia W Morch CD Andersen OK. Exploration of the conditioning electrical stimulation frequencies for induction of long-term potentiation-like pain amplification in humans. Exp Brain Res. (2016) 234:2479–89. doi: 10.1007/s00221-016-4653-1, PMID: 27093867

[3] Klein T Magerl W Hopf HC Sandkuhler J Treede RD. Perceptual correlates of nociceptive long-term potentiation and long-term depression in humans. J Neurosci. (2004) 24:964–71. doi: 10.1523/JNEUROSCI.1222-03.2004, PMID: 14749441 PMC6729815

[4] Xia W Morch CD Andersen OK. Test-retest reliability of 10 Hz conditioning electrical stimulation inducing long-term potentiation (LTP)-like pain amplification in humans. PLoS One. (2016) 11:e0161117. doi: 10.1371/journal.pone.0161117, PMID: 27529175 PMC4986952

[5] van den Broeke EN van Heck CH Ceelen LA van Rijn CM van Goor H Wilder-Smith OH. The effect of high-frequency conditioning stimulation of human skin on reported pain intensity and event-related potentials. J Neurophysiol. (2012) 108:2276–81. doi: 10.1152/jn.00391.2012, PMID: 22855779

[6] Enax-Krumova E Attal N Bouhassira D Freynhagen R Gierthmuhlen J Hansson P. Contralateral sensory and pain perception changes in patients with unilateral neuropathy. Neurology. (2021) 97:e389–402. doi: 10.1212/WNL.0000000000012229, PMID: 34011572

[7] Konopka KH Harbers M Houghton A Kortekaas R van Vliet A Timmerman W. Bilateral sensory abnormalities in patients with unilateral neuropathic pain; a quantitative sensory testing (QST) study. PLoS One. (2012) 7:e37524. doi: 10.1371/journal.pone.0037524, PMID: 22629414 PMC3358252

[8] van den Broeke EN van Rijn CM Biurrun Manresa JA Andersen OK Arendt-Nielsen L Wilder-Smith OH. Neurophysiological correlates of nociceptive heterosynaptic long-term potentiation in humans. J Neurophysiol. (2010) 103:2107–13. doi: 10.1152/jn.00979.2009, PMID: 20164395

[9] van den Broeke EN Urdi M Mouraux A Biurrun Manresa JA Torta DME. High-frequency electrical stimulation of cutaneous nociceptors differentially affects pain perception elicited by homotopic and heterotopic electrical stimuli. J Neurophysiol. (2021) 126:1038–44. doi: 10.1152/jn.00289.2021, PMID: 34432997

[10] van den Broeke EN Vanmaele T Mouraux A Stouffs A Biurrun-Manresa J Torta DM. Perceptual correlates of homosynaptic long-term potentiation in human nociceptive pathways: a replication study. R Soc Open Sci. (2021) 8:200830. doi: 10.1098/rsos.200830, PMID: 33614062 PMC7890496

[11] van den Broeke EN van Heck CH van Rijn CM Wilder-Smith OH. Neural correlates of heterotopic facilitation induced after high frequency electrical stimulation of nociceptive pathways. Mol Pain. (2011) 7:28. doi: 10.1186/1744-8069-7-28 21507241 PMC3108312

[12] Henrich F Magerl W Klein T Greffrath W Treede RD. Capsaicin-sensitive C- and A-fibre nociceptors control long-term potentiation-like pain amplification in humans. Brain. (2015) 138:2505–20. doi: 10.1093/brain/awv108 25943423

[13] Klein T Stahn S Magerl W Treede RD. The role of heterosynaptic facilitation in long-term potentiation (LTP) of human pain sensation. Pain. (2008) 139:507–19. doi: 10.1016/j.pain.2008.06.001, PMID: 18602755

[14] Lang S Klein T Magerl W Treede RD. Modality-specific sensory changes in humans after the induction of long-term potentiation (LTP) in cutaneous nociceptive pathways. Pain. (2007) 128:254–63. doi: 10.1016/j.pain.2006.09.026, PMID: 17123732

[15] van den Broeke EN Mouraux A. High-frequency electrical stimulation of the human skin induces heterotopical mechanical hyperalgesia, heat hyperalgesia, and enhanced responses to nonnociceptive vibrotactile input. J Neurophysiol. (2014) 111:1564–73. doi: 10.1152/jn.00651.2013, PMID: 24453277

[16] Kronschlager MT Drdla-Schutting R Gassner M Honsek SD Teuchmann HL Sandkuhler J. Gliogenic LTP spreads widely in nociceptive pathways. Science. (2016) 354:1144–8. doi: 10.1126/science.aah5715, PMID: 27934764 PMC6145441

[17] Ruscheweyh R Wilder-Smith O Drdla R Liu XG Sandkuhler J. Long-term potentiation in spinal nociceptive pathways as a novel target for pain therapy. Mol Pain. (2011) 7:20. doi: 10.1186/1744-8069-7-20 21443797 PMC3078873

[18] Sandkuhler J. Translating synaptic plasticity into sensation. Brain. (2015) 138:2463–4. doi: 10.1093/brain/awv193 26304147

[19] van den Broeke EN Geene N van Rijn CM Wilder-Smith OH Oosterman J. Negative expectations facilitate mechanical hyperalgesia after high-frequency electrical stimulation of human skin. Eur J Pain. (2014) 18:86–91. doi: 10.1002/j.1532-2149.2013.00342.x, PMID: 23754275

[20] Della Porta D Vilz ML Kuzminova A Filbrich L Mouraux A Legrain V. No evidence for an effect of selective spatial attention on the development of secondary hyperalgesia: a replication study. Front Hum Neurosci. (2022) 16:997230. doi: 10.3389/fnhum.2022.997230, PMID: 36405082 PMC9670179

[21] World Medical Association. World medical association declaration of Helsinki: ethical principles for medical research involving human subjects. JAMA. (2013) 310:2191–4. doi: 10.1001/jama.2013.281053 24141714

[22] Jensen MPTJ Romano JM Fisher LD. Comparative reliability and validity of chronic pain intensity measures. Pain. (1999) 83:157–62. doi: 10.1016/S0304-3959(99)00101-3 10534586

[23] Hansen N Klein T Magerl W Treede RD. Psychophysical evidence for long-term potentiation of C-fiber and Adelta-fiber pathways in humans by analysis of pain descriptors. J Neurophysiol. (2007) 97:2559–63. doi: 10.1152/jn.01125.2006, PMID: 17215503

[24] Schilder A Magerl W Hoheisel U Klein T Treede RD. Electrical high-frequency stimulation of the human thoracolumbar fascia evokes long-term potentiation-like pain amplification. Pain. (2016) 157:2309–17. doi: 10.1097/j.pain.0000000000000649, PMID: 27322440

[25] Rupp R Biering-Sorensen F Burns SP Graves DE Guest J Jones L. International standards for neurological classification of spinal cord injury: revised 2019. Top Spinal Cord Inj Rehabil. (2021) 27:1–22. doi: 10.46292/sci2702-1, PMID: 34108832 PMC8152171

[26] Van der Cruyssen F Van Tieghem L Croonenborghs TM Baad-Hansen L Svensson P Renton T. Orofacial quantitative sensory testing: current evidence and future perspectives. Eur J Pain. (2020) 24:1425–39. doi: 10.1002/ejp.1611, PMID: 32557971 PMC7497080

[27] Reimer M Forstenpointner J Hartmann A Otto JC Vollert J Gierthmühlen J. Sensory bedside testing: a simple stratification approach for sensory phenotyping. Pain Rep. (2020) 5:e820. doi: 10.1097/PR9.0000000000000820, PMID: 32903958 PMC7447375

[28] Kirshblum S Didesch M Botticello A Kong B Androwis D. Patient preferences for order of the sensory portion of the international standards for neurological classification of spinal cord injury (ISNCSCI) examination. J Spinal Cord Med. (2019) 42:719–24. doi: 10.1080/10790268.2019.1582602, PMID: 30888263 PMC6830224

[29] Perez C Galvez R Huelbes S Insausti J Bouhassira D Diaz S. Validity and reliability of the Spanish version of the DN4 (Douleur Neuropathique 4 questions) questionnaire for differential diagnosis of pain syndromes associated to a neuropathic or somatic component. Health Qual Life Outcomes. (2007) 5:66. doi: 10.1186/1477-7525-5-66, PMID: 18053212 PMC2217518

[30] Kroenke K Spitzer RL Williams JB. The PHQ-9: validity of a brief depression severity measure. J Gen Intern Med. (2001) 16:606–13. doi: 10.1046/j.1525-1497.2001.016009606.x, PMID: 11556941 PMC1495268

[31] Muñoz-Navarro R Cano-Vindel A Medrano LA Schmitz F Ruiz-Rodriguez P Abellan-Maeso C. Utility of the PHQ-9 to identify major depressive disorder in adult patients in Spanish primary care centres. BMC Psychiatry. (2017) 17:291. doi: 10.1186/s12888-017-1450-8, PMID: 28793892 PMC5550940

[32] Muñoz-Navarro R Cano-Vindel A Moriana JA Medrano LA Ruiz-Rodriguez P Aguero-Gento L. Screening for generalized anxiety disorder in Spanish primary care centers with the GAD-7. Psychiatry Res. (2017) 256:312–7. doi: 10.1016/j.psychres.2017.06.023 28666201

[33] Spitzer RL Kroenke K Williams JB Lowe B. A brief measure for assessing generalized anxiety disorder: the GAD-7. Arch Intern Med. (2006) 166:1092–7. doi: 10.1001/archinte.166.10.1092 16717171

[34] Rios-Leon M Valera-Calero JA Ortega-Santiago R Varol U Fernandez-de-Las-Penas C Plaza-Manzano G. Analyzing the interaction between clinical, neurophysiological and psychological outcomes underlying chronic plantar heel pain: a network analysis study. Int J Environ Res Public Health. (2022) 19:10301. doi: 10.3390/ijerph191610301, PMID: 36011936 PMC9408584

[35] van Hout B Janssen MF Feng YS Kohlmann T Busschbach J Golicki D. Interim scoring for the EQ-5D-5L: mapping the EQ-5D-5L to EQ-5D-3L value sets. Value Health. (2012) 15:708–15. doi: 10.1016/j.jval.2012.02.008, PMID: 22867780

[36] van den Broeke EN Mouraux A. Enhanced brain responses to C-fiber input in the area of secondary hyperalgesia induced by high-frequency electrical stimulation of the skin. J Neurophysiol. (2014) 112:2059–66. doi: 10.1152/jn.00342.2014, PMID: 25098966 PMC4274930

[37] van den Broeke EN Lambert J Huang G Mouraux A. Central sensitization of mechanical nociceptive pathways is associated with a long-lasting increase of pinprick-evoked brain potentials. Front Hum Neurosci. (2016) 10:531. doi: 10.3389/fnhum.2016.00531, PMID: 27812331 PMC5071355

[38] Pieretti S Di Giannuario A Di Giovannandrea R Marzoli F Piccaro G Minosi P. Gender differences in pain and its relief. Ann Ist Super Sanita. (2016) 52:184–9. doi: 10.4415/ANN_16_02_09, PMID: 27364392

[39] Klein T Magerl W Treede RD. Perceptual correlate of nociceptive long-term potentiation (LTP) in humans shares the time course of early-LTP. J Neurophysiol. (2006) 96:3551–5. doi: 10.1152/jn.00755.2006, PMID: 17021023

[40] Ji G Zhou S Carlton SM. Intact Adelta-fibers up-regulate transient receptor potential A1 and contribute to cold hypersensitivity in neuropathic rats. Neuroscience. (2008) 154:1054–66. doi: 10.1016/j.neuroscience.2008.04.039, PMID: 18514429 PMC2530901

[41] Mackenzie RA Burke D Skuse NF Lethlean AK. Fibre function and perception during cutaneous nerve block. J Neurol Neurosurg Psychiatry. (1975) 38:865–73. doi: 10.1136/jnnp.38.9.865, PMID: 1185225 PMC492115

[42] Treede RD Meyer RA Raja SN Campbell JN. Peripheral and central mechanisms of cutaneous hyperalgesia. Prog Neurobiol. (1992) 38:397–421. doi: 10.1016/0301-0082(92)90027-C 1574584

[43] Luz LL Lima S Fernandes EC Kokai E Gomori L Szucs P. Contralateral afferent input to lumbar lamina I neurons as a neural substrate for mirror-image pain. J Neurosci. (2023) 43:3245–58. doi: 10.1523/JNEUROSCI.1897-22.2023, PMID: 36948583 PMC10162462

[44] de la Llave-Rincon AI Fernandez-de-las-Penas C Fernandez-Carnero J Padua L Arendt-Nielsen L Pareja JA. Bilateral hand/wrist heat and cold hyperalgesia, but not hypoesthesia, in unilateral carpal tunnel syndrome. Exp Brain Res. (2009) 198:455–63. doi: 10.1007/s00221-009-1941-z, PMID: 19618171

[45] van den Broeke EN Lenoir C Mouraux A. Secondary hyperalgesia is mediated by heat-insensitive A-fibre nociceptors. J Physiol. (2016) 594:6767–76. doi: 10.1113/JP272599, PMID: 27377467 PMC5108905

[46] De R Maihofner C. Centrally mediated sensory decline induced by differential C-fiber stimulation. Pain. (2008) 138:556–64. doi: 10.1016/j.pain.2008.02.005, PMID: 18358612

[47] Kim HY Jun J Wang J Bittar A Chung K Chung JM. Induction of long-term potentiation and long-term depression is cell-type specific in the spinal cord. Pain. (2015) 156:618–25. doi: 10.1097/01.j.pain.0000460354.09622.ec, PMID: 25785524 PMC4365505

[48] Huang CC Hsu KS. Progress in understanding the factors regulating reversibility of long-term potentiation. Rev Neurosci. (2001) 12:51–68. doi: 10.1515/revneuro.2001.12.1.51, PMID: 11236065

